# Thalidomide Attenuates Epileptogenesis and Seizures by Decreasing Brain Inflammation in Lithium Pilocarpine Rat Model

**DOI:** 10.3390/ijms24076488

**Published:** 2023-03-30

**Authors:** Irán M. Cumbres-Vargas, Sergio R. Zamudio, Luz A. Pichardo-Macías, Eduardo Ramírez-San Juan

**Affiliations:** Departamento de Fisiología, Escuela Nacional de Ciencias Biológicas, Instituto Politécnico Nacional, Mexico City 07738, Mexico; iranmcv94@gmail.com (I.M.C.-V.); szamudio@ipn.mx (S.R.Z.)

**Keywords:** thalidomide, antiepileptogenic, anti-ictogenic, neuroinflammation, temporal lobe epilepsy

## Abstract

Thalidomide (TAL) has shown potential therapeutic effects in neurological diseases like epilepsy. Both clinical and preclinical studies show that TAL may act as an antiepileptic drug and as a possible treatment against disease development. However, the evidence for these effects is limited. Therefore, the antiepileptogenic and anti-inflammatory effects of TAL were evaluated herein. Sprague Dawley male rats were randomly allocated to one of five groups (n = 18 per group): control (C); status epilepticus (SE); SE-TAL (25 mg/kg); SE-TAL (50 mg/kg); and SE-topiramate (TOP; 60mg/kg). The lithium-pilocarpine model was used, and one day after SE induction the rats received pharmacological treatment for one week. The brain was obtained, and the hippocampus was micro-dissected 8, 18, and 28 days after SE. TNF-α, IL-6, and IL-1β concentrations were quantified. TOP and TAL (50 mg/kg) increased the latency to the first of many spontaneous recurrent seizures (SRS) and decreased SRS frequency, as well as decreasing TNF-α and IL-1β concentrations in the hippocampus. In conclusion, the results showed that both TAL (50 mg/kg) and TOP have anti-ictogenic and antiepileptogenic effects, possibly by decreasing neuroinflammation.

## 1. Introduction

Temporal lobe epilepsy (TLE) is the most common epileptic syndrome in adults [1,2]; it is characterized by focal seizures originating from one of the temporal lobes [3,4]. TLE usually develops secondary to an initial brain insult such as trauma, infection, or status epilepticus (SE). The initial insult is followed by a latent period (epileptogenesis), after which spontaneous recurrent seizures (SRS) occur [5]. Although there has been significant progress in research and treatment over the past few decades, epilepsy still affects approximately 65 million people globally [6], and 30% of epileptic patients are refractory to pharmacological therapies [7]. In addition, there are some concerns about current epilepsy treatments. First, existing medications do not address all comorbidities that occur in epileptic patients, for example, neuroinflammation and neuronal loss [8,9], and, unfortunately, no medication has been shown to prevent epilepsy in people at risk or modify disease progression [10]. Therefore, it is necessary to find new antiepileptic and/or antiepileptogenic drugs to treat the disease [11,12,13].

Epileptogenesis is a dynamic process that transforms a healthy brain into a tissue capable of generating SRS, resulting in an epileptic condition and/or the progression of epilepsy after it is established (often termed “secondary epileptogenesis”) [14,15]. This period starts when the injury has been produced and ends when the first SRS appears [16,17,18]. This offers a window of opportunity following acute brain insults that allows intervention with preventive treatment [19,20]. There is evidence that neuroinflammation plays a key part in epileptogenesis [21,22]. In turn, it affects other processes such as neurogenesis and leads to abnormal reorganization of neuronal networks, mainly in the hippocampus, further promoting neuronal death [23,24,25]. Consequently, inhibiting neuroinflammation may be effective for the reduction or even prevention of epileptogenesis. In animal models of acquired epilepsy, several drugs have been shown to have antiepileptogenic properties or disease-modifying effects, as well as anti-inflammatory properties. These promising agents include atorvastatin, ceftriaxone, topiramate (TOP) [20], levetiracetam [26], and thalidomide (TAL) [27] among others.

TAL was synthesized as a sedative and antiemetic drug in 1950. However, due to its high teratogenicity incidence, it was withdrawn [28]. In 1965, it was observed that patients with leprosy erythema nodosum treated with TAL had a significant health improvement [29]. Thus, the Drug and Food Administration (FDA) approved the use of TAL for leprosy in 1998, and in 2006, it was approved for the treatment of multiple myeloma [30]. Regarding epilepsy, TAL has shown antiepileptic effects in different animal models [27,31,32] and in patients with refractory epilepsy [33]. Although its possible antiepileptic mechanism of action is not completely understood, it has been closely associated with immunomodulatory and neuromodulatory actions [30]. The inhibition of tumor necrosis factor alpha (TNF α) [34,35], nitric oxide signaling, N-methyl-D-aspartate acid receptor (NMDAR), cyclooxygenases (COXs), and opiopeptidergic transmission by TAL may contribute to its anti-seizure activity [32,36,37].

Herein, the possible antiepileptic effect of TAL was evaluated using an animal model of TLE induced by lithium and pilocarpine in rats. In addition, whether this property is due to an anti-inflammatory effect in the hippocampus was investigated.

## 2. Results

### 2.1. Antiepileptogenic Effect of Thalidomide

**Figure 1:**
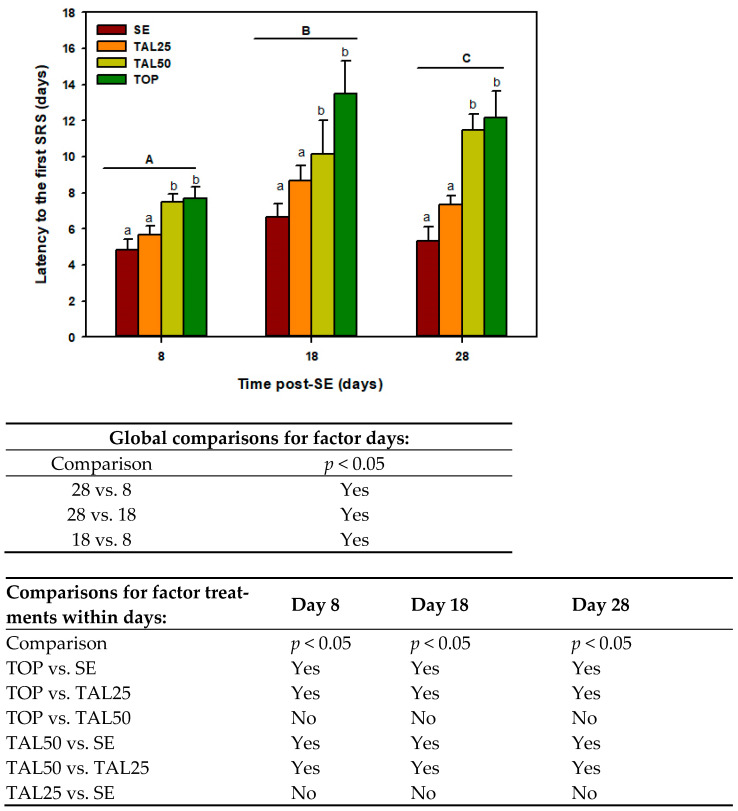
Effect of thalidomide (TAL) and topiramate (TOP) on the latency to the first spontaneous recurrent seizure observed 8, 18 and 28 days after status epilepticus (SE). Each bar represents the mean + S.E.M. of 6 animals per group. Different capital letters denote global comparisons *p* ≤ 0.05 among times, lowercase letters designate *p* ≤ 0.05 among treatments within times, two-way ANOVA followed by Student-Newman-Keuls post-hoc test. The tables show the results of statistical analysis.

Figure 1 illustrates the effect of treatment with TOP (60 mg/kg) or different TAL doses (25 and 50 mg/kg, i.p.) on the latency to the first SRS. A SRS was scored when rats reached ≥4 in Racine scale (tonic clonic bilateral seizures) [38]. A two-way ANOVA revealed a significant effect of pharmacological treatments (F_(3, 60)_ = 13.78, *p* ≤ 0.001) and evaluation time (F_(2, 60)_ = 12.20, *p* ≤ 0.001), but the interaction between the two factors (F_(8, 60)_ = 1.75, *p* = 0.106) was not significant; therefore, global comparisons were made. The post-hoc analysis showed an increased latency to the first convulsion in the TAL50 and TOP groups compared with the SE group at all periods (8, 18, and 28 days). In contrast, no significant change in the latency to the first SRS was found for TAL at a dose of 25 mg/kg. With respect to the evaluation time, the global latency to the first SRS was lower at day 8, greater at day 18, and intermediate at day 28. The SE group had the shortest latency to the first SRS at all times evaluated (4.8 ± 0.60; 6.60 ± 0.71 and 5.33 ± 0.80 days). In this study, one rat died as a result of SE and was replaced.

### 2.2. Anti-ictogenic Effect of Thalidomide

A two-way ANOVA showed significant differences in the number of seizures for treatment (F_(3, 57)_ = 14.88) and post-SE time (F_(3, 60)_ = 20.88); however, the interaction between factors (F_(3, 60)_ =1.18) was not significantly different, thus global comparisons were made. For seizure duration, the analysis showed a significant difference for treatment (F_(3, 57)_ = 31.78), time post-SE (F_(3, 60)_ = 16.99) and the interaction between them (F_(3, 60)_ = 3.94), thus simple main effects were analyzed.

An anticonvulsant effect of TAL was observed; it decreased the duration of epileptic seizures compared with the SE group on days 18 and 28. It also reduced the SRS number during all periods of evaluation (8, 18, and 28 post-SE day). The number and duration of epileptic seizures increased significantly from days 8 to 28 post-SE in all groups (Table 1). It is important to note that the number and duration of seizures were expected to increase given more days of observation. Indeed, this is the case for the SE group, where the total number of seizures significantly increases during the temporal disease course. However, both TAL doses (25 mg/kg and 50 mg/kg) and TOP (60 mg/kg) significantly reduced SRS number and duration.

### 2.3. Anti-Inflammatory Effect of Thalidomide

**Figure 2:**
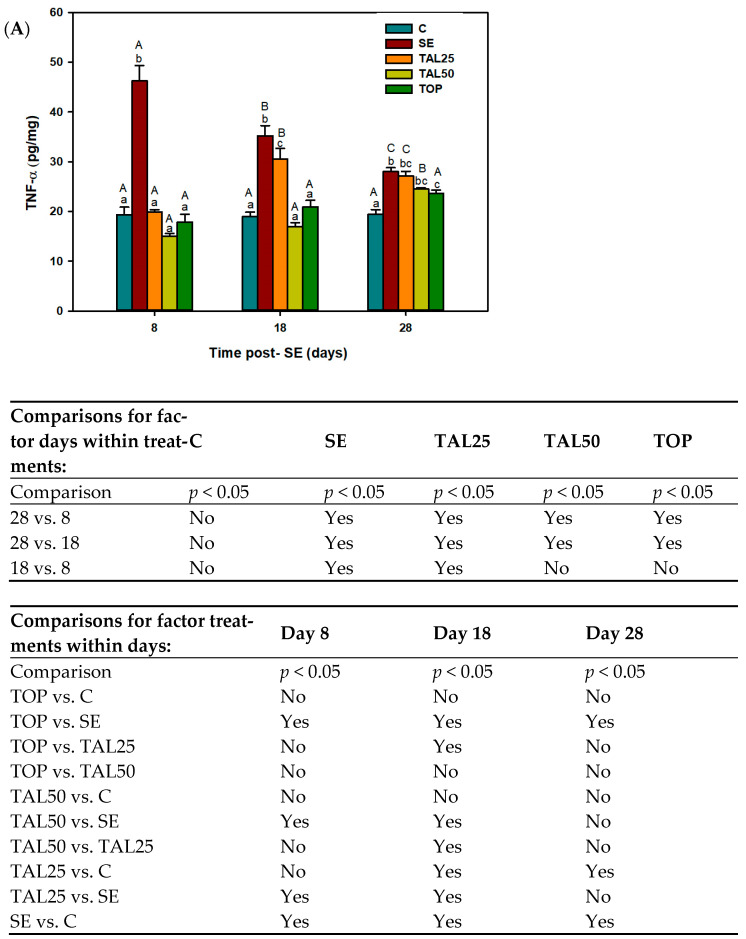

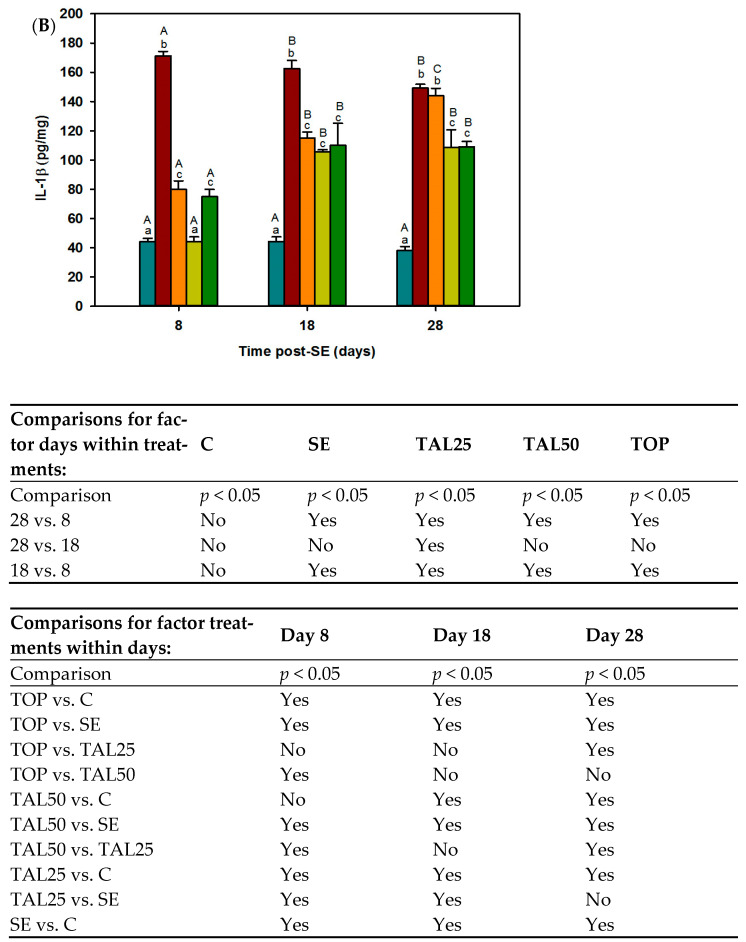

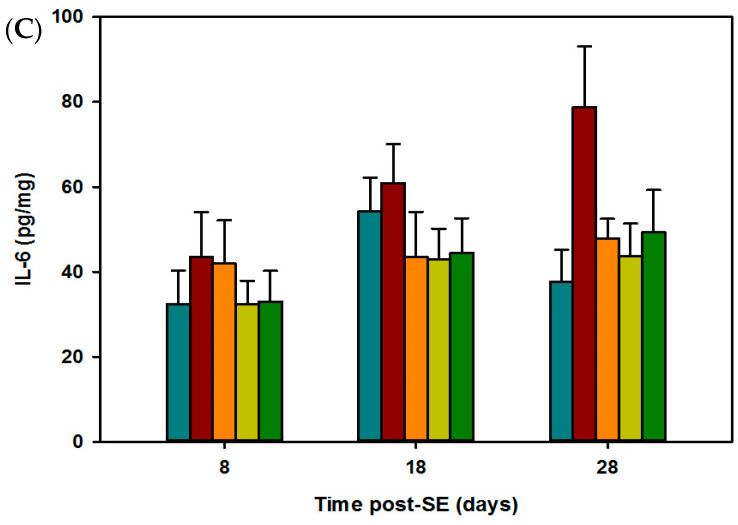
Effect of thalidomide (TAL) and topiramate (TOP) on hippocampal proinflammatory cytokines quantified 8, 18 and 28 days after status epilepticus (SE). (**A**) TNF-α concentration of (**B**) IL-1β concentration and (**C**) IL-6 concentration. Each bar represents the mean + S.E.M. of 6 animals per group. Different capital letters denote *p* ≤ 0.05 among times within treatments, lowercase letters designate *p* ≤ 0.05 among treatments within times, two-way ANOVA followed by the Student-Newman-Keuls post-hoc test. C, control group. The tables show the results of statistical analysis.

In order to explore the anti-inflammatory activity of TAL, the hippocampal concentrations of TNF-α, IL-6, and IL-1β was quantified. The results are shown in Figure 2. A two-way ANOVA revealed that both treatment and time lowered TNF-α concentration in the hippocampus. Since their interaction was significant (F_(4, 75)_ = 15.63), simple main effects were analyzed. TNF-α levels were approximately 20 pg/mg in the C and TOP groups, and they remained at that value during the 3 periods evaluated (8, 18 and 28 post-SE). In the SE group, TNF-α concentration was 46.22 pg/mg on day 8, and it decreased significantly to 35.23 and 28 pg/mg on days 18 and 28, respectively. In the TAL25 group, TNF-α concentration was 20 pg/mg on day 8, increasing significantly at day 18 (30.51 pg/mg) and 28 (27.11 pg/mg). Regarding TAL treatment (50 mg/kg), TNF-α concentration on day 8 and 18 was around 15 pg/mg, it increased significantly to 25 pg/mg on day 28. On day 8, TNF-α concentration in the SE group increased significantly. Pharmacological treatment with TAL or TOP significantly returned TNF-α levels to control values, showing a protective effect against the increment induced by SE. On day 18, TNF-α concentration is still increased in the SE group; 25 mg/kg TAL no longer protects against this increase, while treatment with a higher TAL dose or TOP lowered TNF-α concentration (it showed a similar value to controls). Finally, at day 28, only the protective effect of TOP was observed (Figure 2A).

IL-1β levels were approximately 44 pg/mg in the C group and they remained at that value in the 3 periods evaluated (8-, 18-, and 28-days post-SE). In the SE group, IL-1β levels were 171.24, 162.49 and 149.26 pg/mg on days 8, 18 and 28, respectively. IL-1β concentration was 80 pg/mg in rats treated with TAL (25 mg/kg) at day 8, 115 pg/mg on day 18 and 144 pg/mg on day 28. Rats treated with 50 mg/kg TAL showed a similar IL-1β concentration to the C group (44.16 pg/mg) at day 8; however, IL-1β increased to 105 and 109 pg/mg on days 18 and 28, respectively. IL-1β concentration in the TOP group was 74.91 pg/mg at day 8 and 109 pg/mg on days 18 and 28. IL-1β concentration increased significantly on days 8, 18 and 28 in the SE group compared with C rats. On the same day of evaluation, all treatments (TAL and TOP) showed a protective effect against the IL-1β increment induced by SE. These treatments significantly decreased the levels of this proinflammatory cytokine. It is important to note that the TAL50 group showed values similar to the C group. On day 18, TAL25, TAL50 and TOP decreased IL-1β concentration with respect to the SE group; however, all these groups presented a higher concentration than the C group.

On day 28, only the TAL50 and TOP groups maintained the IL-1β concentration below the SE group, although the values were far from the values of the C group (Figure 2B). Finally, with respect to IL-6 no significant differences were found (Figure 2C).

**Figure 3:**
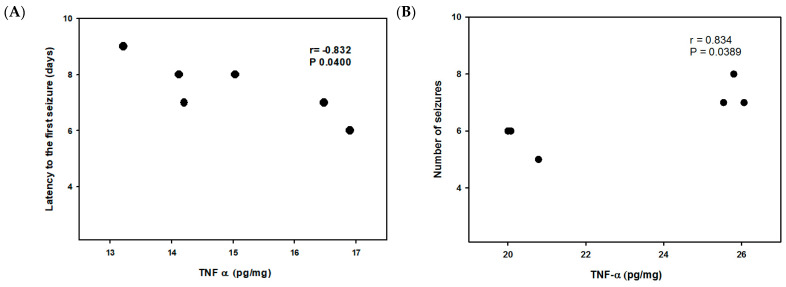

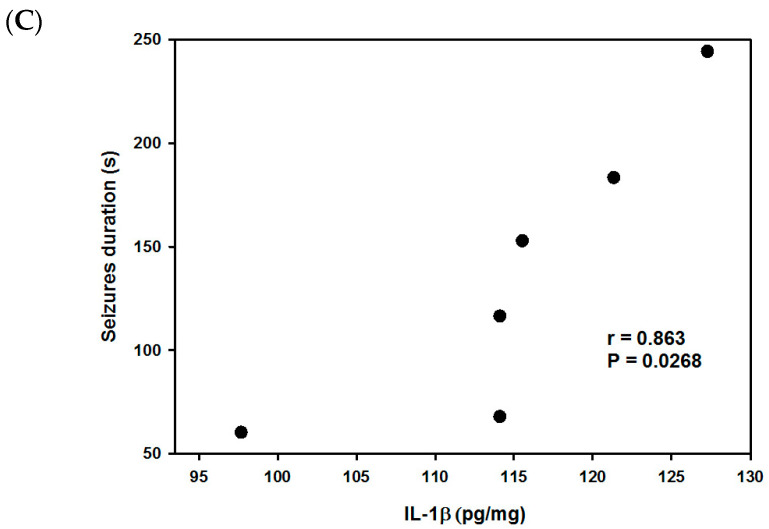
(**A**) Correlation between TNF-α concentration and the latency to the first seizure in the TAL50 group at day 8 post-SE. (**B**) Correlation between TNF-α concentration and the number of SRS in the TOP group at 28 days post-SE. (**C**) Correlation between IL-1β concentration and the duration of SRS in the TAL25 group at 18 days post-SE. *p* < 0.05 Pearson correlation. (n = 6).

Additionally, Pearson correlations were performed among the variables, to know if increases in the hippocampal concentrations of TNF-α, and IL-1β are correlated with the latency to the first seizure or number and duration of seizures. The analysis showed three significant correlations (Figure 3). In the TAL50 group on day 8, the latency to the first seizure and TNF-α concentration were significantly correlated (r = −0.83, *p* = 0.04). It shows that if TNF-α concentration increases, the latency to the first seizure is reduced (Figure 3A). The second correlation was between the number of seizures and TNF-α concentration in the TOP group on day 28 (r = 0.83, *p* = 0.04, Figure 3B). It shows that if TNF-α concentration increases, the number of seizures also increases. The third correlation was between the duration of seizures and IL-1β concentration in the TAL25 group on day 18 (r = 0.86, *p* = 0.03; Figure 3C). This indicates that if IL-1β concentration increases, the seizure duration increases too.

## 3. Discussion

Despite the wide range of pharmacological options to treat epilepsy, most drugs focus exclusively on preventing or suppressing seizures, which are the final product of epileptic process [5]. Recent experimental data in rodents indicates that immediate use of certain anti-seizure drugs after SE or trauma reduces the risk of presenting epilepsy and other alterations related to this disease; thus, these drugs are known as antiepileptogenic drugs [20] and offer the opportunity for disease prevention. Additionally, many studies indicated that inflammation plays an important role in the initiation and maintenance of epileptogenesis and epilepsy [39,40,41,42]. This has raised the question of whether drugs with anti-inflammatory activity could constitute new therapies for the treatment of epilepsy and/or its development (epileptogenesis). As far we know, herein is the first time that antiepileptogenic and anti-ictogenic effects of TAL in a chronic animal model of epilepsy have been describe. Similarly, herein a novel finding is that anti-inflammatory properties of TAL are related to anti-ictogenic and anti-epileptogenic effects.

Our results show that one-week treatment with TAL (50 mg/kg) increases the onset of the first seizure, suggesting potential antiepileptogenic activity. Furthermore, TAL showed anti-ictogenic effects during the chronic disease phase since it decreased both the number and duration of SRS. There are few studies that support the effect of TAL as an “antiepileptic drug”. For example, TAL significantly decreased the frequency and intensity of seizures in patients with Rasmussen syndrome; a syndrome characterized by CNS inflammation [43]. Similar results were reported in a clinical study on patients with refractory epilepsy [33]. In preclinical studies, TAL significantly decreased acute seizures induced by both pentylenetetrazol (PTZ) [31,44] and pilocarpine in mice [32] and rats; additionally, TAL showed protection of the CA1 hippocampal region [45].

TAL has multiple mechanisms of action; thus, the exact mechanisms underlying its antiseizure effects remain elusive. For instance, it has been reported that TAL can act as a neuroprotective drug by activating the PI3K/Akt signaling pathway [46], which is critical for cell survival and growth. Another mechanism may be an increase in inhibitory transmission by TAL interaction with the allosteric site of the GABA_A_ receptor. Docking simulations have shown that TAL and its analogs act on this receptor [47,48] and may cause a possible antagonism to PTZ, based on the effectiveness of TAL in this animal model [32]. Recently, a study demonstrated that TAL exerted a dose-dependent anticonvulsant effect against clonic seizures induced by PTZ in mice. The authors suggested that opiopeptidergic transmission and its interaction with neuronal NO signaling may contribute to the anti-seizure activity of TAL [44].

The results showed that SE significantly enhanced the level of hippocampal proinflammatory cytokines at all evaluation times compared with the control group, particularly TNF-α and IL-1β. This confirms that inflammation participates in the development of epilepsy. This agrees with other works, where it was established that there is a four-fold increase above baseline values in the concentration of proinflammatory cytokines [49]. TNF-α can activate the TNFR1 receptor on astrocytes, which mediates triphosphate/adenosine diphosphate (ATP/ADP) release. This, in turn, activates P2Y1 purinergic receptors, causing an increase in intracellular Ca^2+^, resulting in glutamate release that activates pre and post synaptic NMDA neuronal receptors to increase neuronal excitability [50,51]. TNF-α also up-regulates microglial glutaminase, inducing glutamate release from gap junction CX32 hemi-channels, which promotes neuroexcitotoxicity [52]. Several studies have proposed that gap junctions play a key role in the synchronization of neuronal circuits. Hence, it has been postulated that enhanced gap junctional communication underlies the mechanism involved in both the generation and maintenance of seizures [53,54,55]. Studies in human fetal skin fibroblasts and in rat liver epithelial cells show that TAL enhanced the gap junctional, which correlates to a teratogenic effect [56,57]. On this basis, TAL could increase the neuroexcitability; however, our results show the opposite.

**Figure 4:**
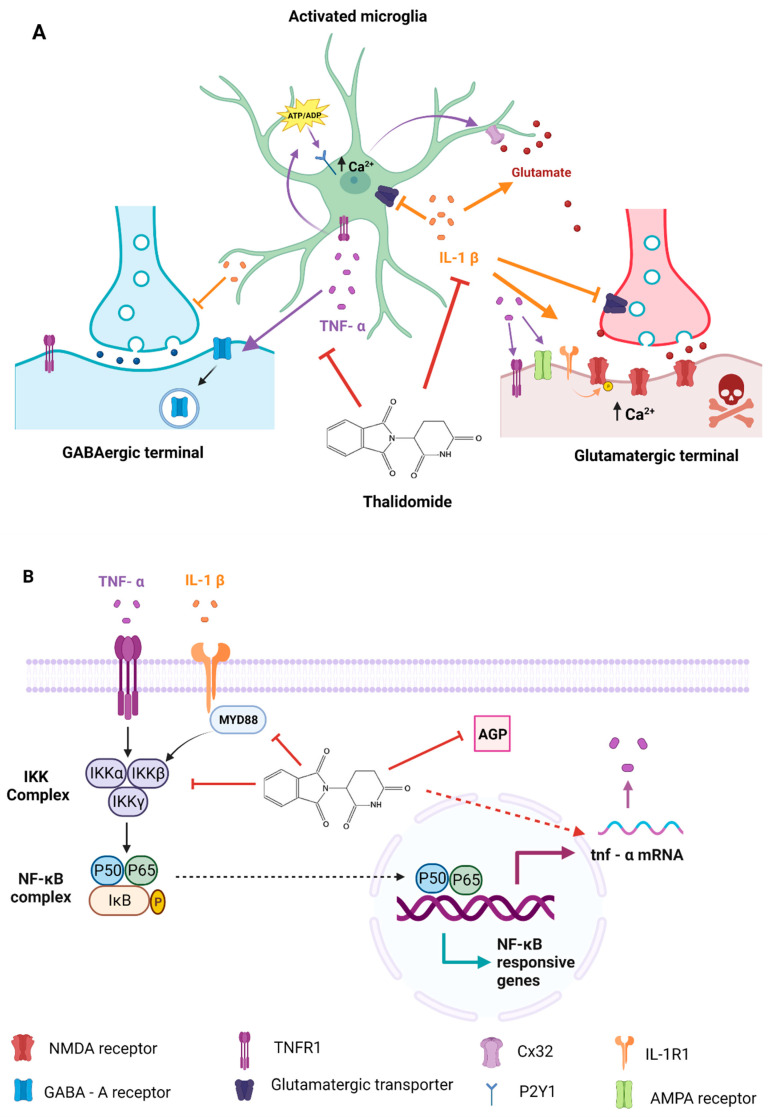
(**A**) Active microglia release proinflammatory cytokines, such as tumor necrosis factor alpha (TNF-α) and interleukin 1 beta (IL-1β). TNF-α induces microglial glutamate release through two mechanisms (1) from gap junction CX32 hemichannels [52] and (2) by activating the TNFR1 receptor in astroglia, which in turn release ATP/ADP to activate the P2Y1 purinergic receptor increasing the intracellular calcium concentration. Glutamate interacting with NMDA receptors promotes neuroexcitotoxicity [50,51]. TNF-α also induces GABA_A_ receptor endocytosis and recruits AMPA receptors lacking GluR2 subunits, a conformation that favors the Ca^2+^ entry, amplifying the glutamate response [58] and activating TNFR1 neuronal receptors inducing cell death. IL-1β promotes NMDA receptor phosphorylation by interacting with its IL-1R1 receptor, which increases intracellular Ca^2+^ and excitotoxicity [75]. IL-1β can also increase glutamate concentrations by inhibiting its recapture by astrocytes and neurons [76,77], as well as by increasing astrocytic release [78,79,80]. Thalidomide (TAL) blocks IL-1β and TNF-α activity, decreasing neuronal damage and inflammation [34,35,73]. (**B**) Putative mechanism of action. TAL blocks TNF-α signaling by destabilizing the 3’unstranslated region (3´UTR) of TNF-α mRNA, inhibiting TNF-α protein synthesis [73]. TAL blocks nuclear factor kappa B (NF-κB) signaling which induces the expression of various pro-inflammatory genes by blocking the IKK complex [34,35] and via myeloid differentiating factor 88 (MyD88) [67]. Finally, TAL also can inhibit TNF-α by blocking α1-acid glycoprotein (AGP) [69]. Orange arrows represent the IL-1β processes while purple arrows represents the TNF-α processes. TAL mechanism are in red arrows. Imagen created with BioRender.com (accessed on 20 February 2023).

On the other hand, TNF-α promotes GABA_A_ receptor endocytosis, decreasing neuronal inhibition [58]. On these bases, inhibition of this cytokine by TAL may help to restore the balance between excitatory and inhibitory systems and explain the reduction in SRS observed herein (Figure 4A).

TAL is considered an immunomodulatory drug with anti-proliferative, anti-inflammatory, and anti-angiogenic activities [59,60]. It can downregulate TNF-α [61,62], IL-6, and IL-1β concentrations [60,63]. In vitro, TAL selectively inhibits TNF-α production of human monocytes stimulated with lipopolysaccharide (LPS) [62]. There are many proposed mechanisms to explain the TNF-α decrease due to TAL. One of them suggests that TAL reduces TNF-α protein synthesis and inflammatory pathways by destabilizing the 3′-untranslated region of TNF-α mRNA [64,65]. Another suggests that reduced NF-κB function decreases TNF-α activity. The NF-κB signaling pathway plays a central role in various immunological responses and has been shown to be a key regulator of inflammatory genes, such as TNF-α and IL-8. NF-κB inhibition is associated with reduced inflammation in animal models [34,35]. TAL blocks NF-κB activation by diminishing IKK complex function, which keeps IkB active in its non-phosphorylated form, which in turn impedes the translocation and activation of NF-κB [66] (Figure 4B). Additionally, it has been reported that TAL inhibits MyD88 (myeloid differentiating factor 88) expression at the RNA and protein levels [67]. MyD88 is an important upstream mediator of NF-kB signaling and is also a key adapter molecule for innate immune response signaling, programmed cell-death, and LPS-induced septic shock syndrome [35] (Figure 4B). Other TNF-α regulators are reactive oxygen species (ROS) [68] and α1-acid glycoprotein (AGP) [69]. Finally, TAL binds to cereblon (CRBN), a protein that functions as a substrate receptor of an E3 ubiquitin ligase complex to degrade proteins [59,70,71], such as the transcription factors IKZF1 (Ikaros) and IKZF3 (Aiolos), which leads to IL-2 upregulation [72,73]. However, there is no evidence to associate CRBN with epilepsy, the anti-inflammatory or anti-ictogenic effects of the drug. In fact, the lack of CRBN in CRBN KO mice does not affect PTZ-induced seizures in these animals [74].

With respect to IL-1β, the interaction with its receptor, IL-1R1, promotes the phosphorylation of the NR2B subunit of the NMDA receptor by *Scr* kinase, which increases intracellular calcium as well as excitotoxicity [75]. IL-1β can also increase glutamate concentrations by both inhibiting astrocytic reuptake [76,77] and increasing astrocytic release either directly or by microglial TNF-α release [78,79,80]. Moreover, in endothelial cells and perivascular astrocytes, IL-1β impairs blood-brain barrier permeability, contributing to inflammation [81]. Finally, several studies show that L-1β concentrations are augmented in TLE, decreasing by 30% GABAergic neurotransmission and contributing to neuronal hyperexcitability [82,83,84]. All these mechanisms contribute to seizure generation [58,85]. They also explain why rats with a significant IL-1β increase show a lower latency to the first SRS (antiepileptogenic effect). TAL treatment, particularly the higher dose (50 mg/kg) at day 8 post-SE, decreases IL-1β concentrations. This could be related to both its anti-ictogenic and antiepileptogenic effects. In accordance, a study using cultures of normal human whole blood stimulated with LPS found that TAL suppressed IL-1β [63].

Surprisingly, IL-6 did not show significant changes in the SE group. In agreement with this finding, no changes in IL-6 concentration were found in the cortex and hippocampus of rats subjected to kindling [86]. In contrast, there are data indicating that this cytokine is increased after SE in the parietal cortex, hippocampus, and amygdala of rats treated with kainic acid [49] and in the serum of patients diagnosed with TLE [87]. TAL decreases IL-6 levels in human lung fibroblasts [88], but otherwise there is no other information about its effects on IL-6, especially in epileptic tissue. Differences in IL-6 levels depend on the type of tissue sample and the moment when the evaluation is completed. Therefore, it is possible that IL-6 could augment along the course of the disease. However, since at the evaluation times of this study IL-6 levels did not change, this suggests that IL-6 did not intervene in the inflammation generated during epileptogenesis.

TOP was originally approved for the treatment of both partial-onset and generalized-onset seizures [89]. However, it has also shown efficacy against several neurological disorders, including pain syndromes, migraine, and treatment for drug-abuse [90,91,92,93]. Our data show that one week of TOP treatment increased the latency to onset of the first epileptic seizure, as well as seizure number and duration of seizures. This agrees with other studies showing that TOP administration after pilocarpine-induced SE was effective in reducing the number of rats that developed epilepsy (>60%) compared with the positive control group [94]. Similar results were reported by Suchomelova et al. (2006) [95]. A neuroprotective effect in the pilocarpine model was also reported; TOP treatment suppressed SE-induced neuronal damage in limbic structures, including the dorsal and ventral hippocampus, basolateral amygdala, and piriform cortex. The authors suggest that neuroprotection is a key mechanism of TOP against SRS and concomitant emotional and cognitive impairments [96]. In contrast, Mazarati et al., (2007) observed that TOP failed to block epileptogenesis in a kindling model but exhibited age-dependent disease-modifying effects [97]. The antiepileptic mechanisms proposed for this drug are the blockade of voltage-gated sodium channels [98], GABAergic transmission potentiation via the GABAA receptor [99], and AMPA/KA glutamate receptor antagonism [100]. The overall effect is to decrease excitatory activity and favor inhibitory activity.

Additionally, in both preclinical and clinical studies, TOP decreased the concentration of various proinflammatory cytokines, such as IL-1β, IFN-ɤ, IL-6, IL-10, IL-17 and TNF-α [101,102]. This data is in accordance with our results showing a decrease in the concentrations of IL-1β and TNF-α in the TOP group. Its anti-inflammatory property has been associated with two of its main mechanisms of action as an antiepileptic drug. The first is by modulating GABA signaling, since GABA may also act as an immunomodulatory molecule that modulates cytokine release [103,104]. The second mechanism involves blockade of voltage-gated sodium channels [104]. For example, phenytoin, an antiepileptic drug that blocks voltage-gated sodium channels, decreases IL-1β and TNF-α release [105]. In addition, has been reported that TOP has anti-apoptotic features [106,107]. Taken together, this information suggests that the anti-inflammatory and anti-apoptotic properties of TOP could be important for its antiepileptic effects.

Inflammation is an important factor for epileptogenesis [108,109,110] and epilepsy. Therefore, anti-inflammatory drugs could be integrated into epilepsy therapy. Indeed, the addition of anti-inflammatory drugs to antiepileptic drugs has been shown to decrease epilepsy-related neurological problems in clinical studies [111,112,113]. Further studies are necessary in order to understand the impact and mechanisms of the inflammatory process during epileptogenesis. The investigation of various anti-inflammatory drugs and antiepileptics that can prevent epilepsy development after an initial precipitating injury should be intensified. This constitutes one of the main challenges in the field of epilepsy.

## 4. Materials and Methods

### 4.1. Animals

Adult male Sprague Dawley rats weighing 260–300 g were used in this study. Rats were maintained individually in acrylic cages under controlled environmental conditions: regulated temperature (22 ± 2 °C), light/dark cycle (12:12 h; lights on 7:00 a.m.) plus food and water available ad libitum. All the experimental procedures were performed in accordance with the Mexican law (SAGARPA NOM-062-Z00-1999) and the NIH Guide for the Care and Use of Laboratory Animals. The protocol was approved by the local ethics committee for animal experimentation (CEI-ENCB-004-2017). All efforts were made to reduce the number of animals used and to minimize their suffering.

### 4.2. Experimental Groups

**Figure 5:**
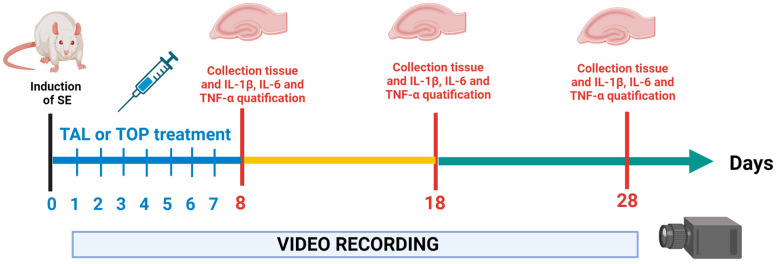
Experimental design. At time 0, status epilepticus (SE) was induced in male Sprague-Dawley rats via administration of lithium-pilocarpine. One day after SE induction rats received daily pharmacological treatment with topiramate (TOP) or thalidomide (TAL) for seven days. One day after treatment the first determination was performed (evaluation time 8). The animals were sacrificed, and hippocampus were collected to determine the concentrations of interleukin IL-1β, IL-6 and necrosis tumoral factor-α (TNF-α) at three different evaluation times: 8, 18 and 28 post-SE days respectively. From 1 to 28 days post-SE, rats were video monitored during 24 h/7 days to observe the latency to the first spontaneous recurrent seizure (SRS) as well as, SRS number and duration.

The animals were randomly allocated to one of the following five groups (n = 18 per group): (1) a control group (C), (2) a group subjected to SE (3) a SE group treated with 25 mg/kg TAL (TAL25) (4) a SE group treated with 50 mg/kg TAL (TAL50) and (5) a SE group treated with 60 mg/kg TOP (TOP is used as a control antiepileptic drug). For the quantification of hippocampal cytokines, the above groups were divided into 3 subgroups according to the day of sacrifice: 8, 18, and 28 post-SE (n = 6 per group, Figure 5).

### 4.3. Status Epilepticus Induction

Animals were administered lithium chloride (127 mg/kg, i.p.; SIGMA, St. Louis, MO, USA) for 19 h before receiving scopolamine methyl-bromide (1 mg/kg, i.p.; SIGMA, St. Louis, MO, USA) [114,115]. Thirty minutes later, the animals were administered pilocarpine hydrochloride (50 mg/kg, i.p.; SIGMA, St. Louis, MO, USA) to induce SE. Convulsions were scored according to the Racine scale (Table 2). The onset of SE was defined as continuous convulsive activity for more than 5 min, reaching stage 4 or 5 on the Racine scale [38].

After 60 min of SE, rats were administered with an intramuscular (i.m.) injection of diazepam (5 mg/kg; PISA, Mexico city, Mexico) to suppress the behavioral seizure and were placed on an ice bed for 1 h to reduce the hyperthermia produced by SE [116,117]. The animals received a second dose of diazepam (5 mg/kg, i.m.; PISA, Mexico city, Mexico) eight hours after receiving the first dose. Finally, the rats received an injection of NaCl 0.9% (5 mL s.c.) for rehydration and were housed overnight in a room at 17 ± 2 °C. Nutritional supplements were provided as a source of food until all the rats returned to eating pellets (~3 days) [118]. Beginning two days after SE, the room temperature was returned at 22 ± 2 °C. To monitor animal health, the experimenters constantly observed them for 8 h after the pilocarpine injection, 3 times a day for an hour for the next 3 days, and daily until the end of the experiment. Control rats only received 0.9% NaCl injections (PISA, Mexico city, Mexico) at each time point.

### 4.4. Spontaneous Recurrent Seizure Monitoring

The rats were placed in individual acrylic cages and continuously (24 h/7 days) video monitored using a camera system (Steren Model CCTV-212, Mexico City) one day after SE induction until 28 days later (Figure 5). Video analysis was performed using the H.264 PlayBack program for Windows (v.1.0.1.15, Infinova, Guangdong, China) to register the latency to the first SRS, as well as the number and duration of seizures. The videos were analyzed by trained blinded observers using the fast-forward speed (eight times normal speed) of the video recorder. Once seizure-like activity was observed, the videotape was rewound to the beginning of the behavior and examined at real-time speed [118]. An animal was considered to have SRS when reaching ≥4 in Racine scale [38]. With these criteria, SRS were considered to be tonic clonic bilateral seizures, as suggested by Goffin et al. 2007. In this classification, stages 1–3 were referred to as focal seizures and grade 4–6 as secondarily generalized seizures [119]. Datasets were generated, recording the latency to the first seizure, as well as the total number and duration of seizures per evaluation period (8, 18 and 28 post-SE days).

### 4.5. Thalidomide and Topiramate Treatment

Drugs were administered i.p. daily for seven days after SE induction (Figure 5). Different doses of TAL (Cayman Chemical, Mexico city, Mexico) were suspended in PBS with 0.5% carboxymethyllcellulose (Sigma-Aldrich St. Louis, MO, USA), while TOP was dissolved in water (PISA, Mexico city, Mexico).

### 4.6. Hippocampal Quantification of TNFα, IL-6 and IL-1β

On days 8, 18, or 28 after SE induction, animal subgroups were killed by decapitation after an anesthetic overdose with sodium pentobarbital (PISABENTAL^®^; 60–90 mg/kg i.p). All efforts were made to minimize animal suffering. After decapitation, the hippocampus was removed [120] and stored at −80 °C until use (Figure 5). To obtained hippocampal homogenates, 50 mg of tissue was homogenized by sonication on ice in 200 µL of lysis buffer: phosphosaline solution 0.05 M, pH 7.4, 0.025 M EDTA, 0.08% Sodium azide 0.05, Triton X-100, protease inhibitor (AEBSF, aprotinin, bestatin, E-64, leupeptin, and pepstatin; ENZO. Homogenates were centrifuged at 12,000× *g* at 4 °C for 20 min. The supernatant was separated and a 1:10 dilution with sample diluent buffer was made for enzyme-linked immunosorbent assay (ELISA) and total protein quantification. The total protein concentration was assessed with bicinconinic acid assays [121].

### 4.7. Enzyme-Linked Immunosorbent Assay

ELISA kits (Sigma-Aldrich, St. Louis, MO, USA) were used to quantify the presence of TNFα, IL-6, and IL-1β in the supernatant of homogenized hippocampi. The kits were used according to manufacturer instructions. Briefly, serial dilutions of protein standards and samples were added to ELISA plates, followed by biotinylated anti-IL-1β, TNFα, and IL-6 antibody addition. Then, a prepared solution of avidin-horseradish peroxidase conjugate complex was added, and the unbound conjugates were washed away with phosphate buffered saline. The reaction was stopped by adding a stopping solution, and absorbance was read at 450 nm.

### 4.8. Statistical Analysis

The results are expressed as the mean ± SEM. Data regarding latency to the first seizure, number of seizures, and duration of generalized seizures were analyzed using two-way analysis of variance (ANOVA) followed by t Student–Newman–Keuls (SNK) post-hoc multiple comparison test. A Pearson correlation of TNF-α, IL-1β, and IL-6 concentrations with respect to the latency to the first seizures, number of seizures, and the duration of generalized seizures was also carried out. A *p* < 0.05 was considered statistically significant. Statistical analysis was performed with Sigma Plot 12.0.

## 5. Conclusions

During epileptogenesis and the chronic phase of epilepsy, the hippocampal concentrations of TFN-α and IL-1β increase on days 8, 18, and 28 post-SE, while the IL-6 concentration did not change at any of these three evaluation times.

TAL and TOP showed an antiepileptogenic and anti-ictogenic effects in rats; in the former case, its effects were dose dependent. TAL reduced TNF-α and IL-1β concentrations in the hippocampus, particularly at the highest dose, on day 8 post-SE. TOP also decreased the concentration of both proinflammatory cytokines at this time. These results suggest that the antiepileptic and antiepileptogenesis effects of these drugs may be related to their anti-inflammatory properties.

## Figures and Tables

**Table 1:** Effect of thalidomide and topiramate on the number and duration of spontaneous recurrent seizures (SRS) observed 8, 18 and 28 days after SE.

Number of Seizures				
TIME POST-SE (Days)	SE	TAL25	TAL50	TOP
8	6.00 ± 0.68 ^a^	4.00 ± 0.63 ^b^	1.66 ± 0.55 ^b^	1.33 ± 0.66 ^b^
18	8.50 ± 1.11 ^a^	5.16 ± 0.87 ^b^	4.00 ± 1.06 ^b^	4.50 ± 1.56 ^b^
28	13.80 ± 1.57 ^a^	8.50 ± 1.52 ^b^	5.80 ± 0.70 ^b^	6.16 ± 0.60 ^b^
**Duration of Seizures (s)**				
8	191.97 ± 30.34	97.20 ± 15.55	32.21 ± 14.69	13.21 ± 10.91
18	311.91 ± 44.6 ^a^	137.56 ± 28.87 ^b^	118.61 ± 31.20 ^b^	69.16 ± 31.27 ^b^
28	636.78 ± 73.58 ^a^	285.15 ± 62.03 ^b^	154.18 ± 19.43 ^b^	119.16 ± 14.99 ^b^

Data represents the mean ± S.E.M. of 6 animals per group. The different lowercase letters denote *p* ≤ 0.05 among treatments within times, two-way ANOVA followed by the Student-Newman-Keuls post-hoc test.

**Table 2:** Racine Scale.

Score	Behavioral Stage
0	No change in behavior
1	Sudden behavior arrest, motionless staring (with orofacial automatism)
2	Head nodding
3	Forelimb clonus with lordotic posture
4	Forelimb clonus with rearing and falling
5	Generalized tonic—clonic activity with loss of postural tone, wild jumping

## Data Availability

The data presented in this study are available on request from the corresponding author.
